# Involvement of DNMT 3B promotes epithelial-mesenchymal transition and gene expression profile of invasive head and neck squamous cell carcinomas cell lines

**DOI:** 10.1186/s12885-016-2468-x

**Published:** 2016-07-08

**Authors:** Li-Hsuen Chen, Wen-Lin Hsu, Yen-Ju Tseng, Dai-Wei Liu, Ching-Feng Weng

**Affiliations:** Department of Life Science and the Institute of Biotechnology, National Dong Hwa University, Hualien, Taiwan; Department of Radiation Oncology, Buddhist Tzu Chi General Hospital, Hualien, Taiwan; School of Medicine, Tzu Chi University, Hualien, Taiwan

**Keywords:** Head and neck cancer, Invasion, EMT, DNMT, miR-29b

## Abstract

**Background:**

The 5-year overall survival rates for head and neck cancer (HNC) relies on distant metastasis. Importantly, the epithelial-mesenchymal transition (EMT) is believed to be an initial step of metastasis. However, the relationship of epigenetic with EMT formation is still unexplored in HNC. This study focuses on invasive subclones of HNC cell lines through the simulation of invasion in vitro; and underlying mechanisms were analyzed including DNA methylation and gene expression profile.

**Methods:**

Invasive subclones of NHC cell lines were successfully obtained using transwell coated with Matrixgel. Cells invaded through 8 μm pore several times were subcultured and examined with EMT features including morphology, EMT marker genes expression, and invasive ability. Moreover, compared the profile of genes expression in parental and invasive cells was analyzed using mRNA expression array.

**Results:**

DNA methyltransferase 3B (DNMT 3B) was upregulated in invasive subclones and might control the 5′ region of E-cadherin (E-cad) methylation and further inhibited E-cad protein expression. Interference of DNMT 3B by siRNA or miRNA 29b could reduce EMT and cell invasion. Expression array analysis revealed the most possible involved pathways in cell invasion including arginine and proline metabolism, TGF-beta, and focal adhesion.

**Conclusions:**

DNMT 3B might control EMT by DNA methylation manner in invasive HNC cell lines. Moreover, miR-29b mimic downregulated DNMT 3B and inhibited EMT and cell invasion indicated the role of therapeutic agent for invasive HNC. Genes identified from array data and new molecules are involved in metastasis of HNC need further validation.

**Electronic supplementary material:**

The online version of this article (doi:10.1186/s12885-016-2468-x) contains supplementary material, which is available to authorized users.

## Background

Head and neck cancer (HNC) is defined as a tumor that develops from mucosal linings of upper aerodigestive tract including the nasopharynx, oral cavity, oropharynx, hypopharynx, and larynx. HNC is the sixth common cancer worldwide [1] and more than 600,000 cases of HNC are reported annually [2]. The major risk factors of HNC are tobacco smoking [3], alcohol consumption [4], chewing of betel quid [5], and human papillomavirus infection [6]. The 5-year overall survival rates for HNC are approximately 50 % but decline to 10 % when metastasis is diagnosed [7, 8]. Hence, the comprehension of the underlying mechanism of HNC metastasis is crucial for therapy and diagnosis of HNC.

Metastasis is believed to consist of four distinct steps including invasion, intravasation, extravasation, and colonization [9]. It is suggested that the acquisition of invasive ability and motility, is the rate-limiting step in the metastatic cascade [9]. Epithelial-mesenchymal transition (EMT) was first recognized as a feature of embryogenesis in the 1980s [10] and involved in many critical cellular process such as embryonic morphogenesis [11], fibrosis [12], and cancer metastasis [13]. There is growing evidentiary support that indicates EMT is an important mechanism for the initial steps of metastasis [10, 14–16]. EMT is categorized by loss of cell polarity, gain spindle-shaped morphology, and enhance cell invasion. The numbers of gene expressions are influenced such as downregulation of epithelia genes including E-cadherin (E-cad), occluding, claudin, cytokeratin, and catenin proteins [10]. Loss of E-cad expression is a hallmark of EMT [13] and often inversely correlated with the tumor stage [17]. Additionally, the upregulation of mesenchymal genes including N-cadherin (N-cad), vimentin, laminin β1 or collagen type VI alpha, as well as various matrix metalloproteinases (MMPs) [18].

Carcinogenesis is a multistep process involved in the accumulation of genetic and epigenetic alterations [19]. Epigenetic changes including DNA methylation, histone modifications, and miRNA-mediated silencing are possibly reversible. This feature makes them attractive targets for diagnostic and therapeutic intervention. Hypermethylated CpG (cytidine-guanosine dinucleotide) islands of tumor suppressor genes are found as a frequent epigenetic marker in human carcinomas [20]. DNA methyltransferases (DNMTs) are enzymes for addition methyl groups to 5′ carbon of the cytosine ring in CpG site. There are three DNMTs in mammalian including DNMT 1, DNMT 3A, and 3B [21]. It is reported that DNMT 1 is responsible for maintenance of parental patterns of DNA methylation and DNMT 3A and 3B establish the new patterns of DNA methylation [22]. The most documented epigenetic control in HNC is the CpG island promoter hypermethylation-related silencing of tumor suppressor genes including p16, DAP-K, RAR beta, MGMT, RASSF1A, and E-cad [23]. These genes are known to function in the cellular pathways involved in cell cycle regulatory, apoptosis, DNA repair, and cell mobility [24].

In the present study, the invasive HNSCC cell lines (A253, RPMI 2650, SCC4, and FaDu) were successfully subcloned from the cells invaded through Matrigel coated transwell several times. The features of EMT in invasive cell lines were assessed including cell morphology and EMT marker genes expression. Our data showed that DNMT 3B was upregulated in invasive subclones and exerted on influence of E-cad methylation. Furthermore, differential gene expressions of invasive subclone were explored using an expression array and processed by DAVID for pathway analysis, which suggests the involvement of possible pathways in cell invasion of HNC.

## Methods

### Cell lines

HNSCC cell lines: A253 (HTB-41™), RPMI 2650 (CCL-30™), SCC4 (CRL1624™), and FaDu (HTB-43™) were obtained from the American Type Culture Collection (ATCC). For maintenance of all HNSCC cell lines, A253 was cultured in McCoy’s 5a Medium, RPMI 2650 in Eagle’s Minimum Essential Medium, SCC4 in DMEM/F12, and FaDu in Minimum Essential Medium, respectively. The cells were cultured in certain medium supplemented with 10 % FBS incubated at 37 °C/5 % CO_2_ atm. The invasive subclones of HNSCC cell lines were obtained as previously described in Chu [25] with minor modification and were named as the generation of the cell lines (A253-3, A253-5, RPMI 2650-8, SCC4-4, and FaDu-8).

### FE-SEM images

The method of taking FE-SEM image followed our lab procedure described in Chang [26] with some minor modifications. In brief, A253-0 and A253-5 (2 × 10^4^) cells were grown on a sterilized indium-tin-oxide (ITO) thin-film deposited on an insulating glass for 16 h. Cells were rinsed, fixed, and dried using a freeze-dry system (LABCONCO FreeZone 4.5). A field-emission scanning electron microscope (FE-SEM, JSM-6500, Japan) was used to examine the cells.

### Transfection of siRNA against DNMT 3B

Plasmid (pSUPER) expressing siRNA against DNMT 3B was obtained from Prof. Show-Li Chen. A stable clone of A253-5si was achieved by selection of G418 after transfection with pSUPER-DNMT 3B. MiRNA 29b mimic was purchased from Ambion (mirVana® miRNA mimic, MC10103). Transfection reagent RNAi Max (Invitrogen) was used for transient transfection miRNA 29b according to the manufacturer’s instructions. Cells transfected with miRNA 29b mimic were cultured for 48 h and applied to the subsequent experiments.

### E-cadherin promoter methylation analysis

A253 and RPMI 2650 cells were treated with 2 μM of 5-aza-2′-deoxycytidine (5′AZA) (Sigma, St Louis, MO)-DNA methyltransferase inhibitors for 4 days and the medium were replaced every 2 days interval. And then cells were harvested for analyze the E-cadherin promoter methylation status with the following methods.

Methylation-specific PCR (MS-PCR) and bisulfite genomic sequencing (BGS) were executed as previously described [27, 28]. Briefly, genomic DNA was isolated from cell lines and applied to bisulfite conversion. The annealing temperature for MS-PCR is 57 °C and for BGS is 62 °C. The amplicons of MS-PCR were visualized and photographed on 2 % agarose gel. The amplicons for BGS were cloned and sequenced. A number of 33 CpG sites were included into BGS analysis. The data were expressed as percentages of methylated CpG sites from five clones. The primer sequences for MS-PCR and BGS of E-cad are listed in Additional file 1: Table S1.

### mRNA and microRNA expression analysis

The methods for analyzing mRNA expression were described previously [28]. Briefly, total RNA isolated from cells was incubated with DNase I and then applied into reverse transcription (RT) reaction. The quantitative PCR (Q-PCR) was executed in triplicate and the relative mRNA expression index was normalized with GADPH using the comparative Ct method (2^-△CT^) [29]. The primer sequences for Q-PCR of gene expression are listed in Additional file 1: Table S1.

For miRNA 29b, TaqMan® Micro Assay Kit was applied. Ten nanograms of total RNA were applied into RT reaction using TaqMan® Reverse Transcription Kit according to the manufacturer’s instructions. The Q-PCR experiment was performed in triplicate and the relative miRNA 29b expression index was normalized with the reference miRNA U6.

### Western blot

The analysis of western blot was described previously [28]. Briefly, total protein was extracted and applied to SDS-polyacrylamide gels for electrophoresis (22 mA per gel). Protein was transferred onto 0.45 μm polyvinylidene difluoride (PVDF) membranes for 1.5 h with 400 mA. The transferred membranes were incubated with blocking buffers for 10 min and then incubated with specific primary antibody against E-cadherin (BD), N-cadherin (Genetex), Vimentin (BD), DNMT 1 (Abcam), DNMT 3A (Abcam), DNMT 3B (Cell signaling), or β-Actin (Cell signaling) at appropriate dilutions at 4 °C overnight. The membranes were washed and incubated with horseradish peroxidase-linked secondary antibody for 1 h at room temperature. Bands were visualization by chemiluminescent reagent and record by photographic film and the intensity of the band was quantified and calculated. The results were conducted independently in triplicate.

### Migration and invasion assay

Migration assay was conducted with ibidi® culture insert. Cell was seeded at both side of the insert (35,000 cells in 70 μl medium) and incubated overnight. The insert was removed next day and photographed at 0 and 48 h. The gap between cells was quantified with Image J and presented as percentage of closure compared to 0 h.

The invasion assay was described previously [28]. Briefly, 100 μl of 80 μg Matrixgel (BD) was previously loaded onto the upper chamber of 24 well transwell (BD, 8 μm pore size) at 37 °C for 2 h and 5 × 10^4^ cells were seeded on the gel (in 200 μl of medium without FBS) and 500 μl of complete medium was added into the lower chamber of the transwell. Cells invaded through transwell were stained after 24 h incubation. Five images were photographed for each transwell under 100X magnification. Cell numbers were counted and calculated.

### Microarray analysis

Total RNA was incubated with DNase I at 37 °C for 15 min and subjected to microarray analysis of mRNA expression delicately using the Human OneArray v6.1 from Phalanx Biotech (Taiwan). Standard selection criteria to identify differentially expressed genes are log_2_ |Fold change| ≥ 1 and *p* < 0.05. Data were processed by using DAVID bioinformatics Resources subjected to KEGG pathway analysis.

### Statistical analysis

Data are presented as the mean ± SD. Statistical analysis between the control and the treatment groups were compared using the Student’s *t*-test. A *p* value < 0.05 was considered statistically significant. * represents *p* < 0.05, ** represent *p* < 0.01, and *** represent *p* < 0.001. Statistical analysis was performed using StatView (version 5.0; SAS Institute, Cary, NC).

## Results

### Morphology changed in invasive subclones of A253

**Fig. 1:**
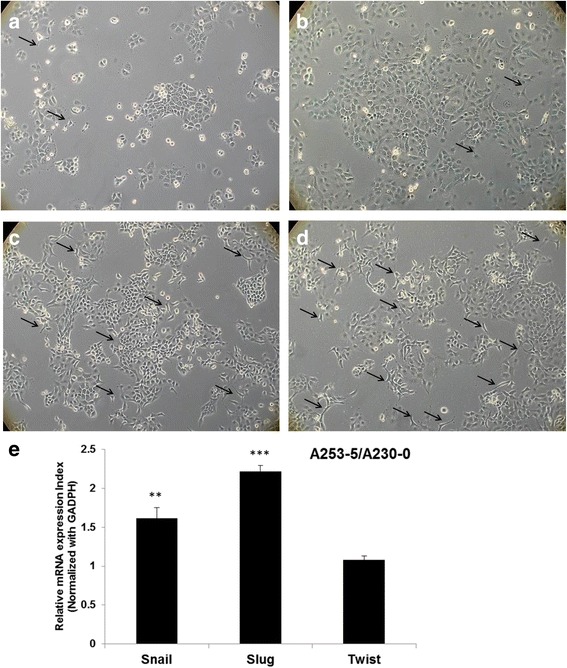
Morphology changed and EMT-related transcription factors expression in invasive subpopulation of A253. Parental A253 (**a** with low density: 4 × 10^3^cells per mm^2^ and **b** with high dansity: 1 × 10^4^ cells per mm^2^) cells were photographed at 100 X magnificence. Invasive subpopulation of A253-3 (**c**) and A253-5 (**d**) (with hight dansity: 1 × 10^4^ cells per mm^2^) had more spindle-shape cells (indicated by *arrows*). **e** Three EMT-related transcription factors (Snail, Slug, and Twist) were evaluated its mRNA expression by Q-PCR. Data was expressed as fold change to A253-0. ** indicated *p* < 0.01 and *** indicated *p* < 0.001 as compared with A253-0

**Fig. 2:**
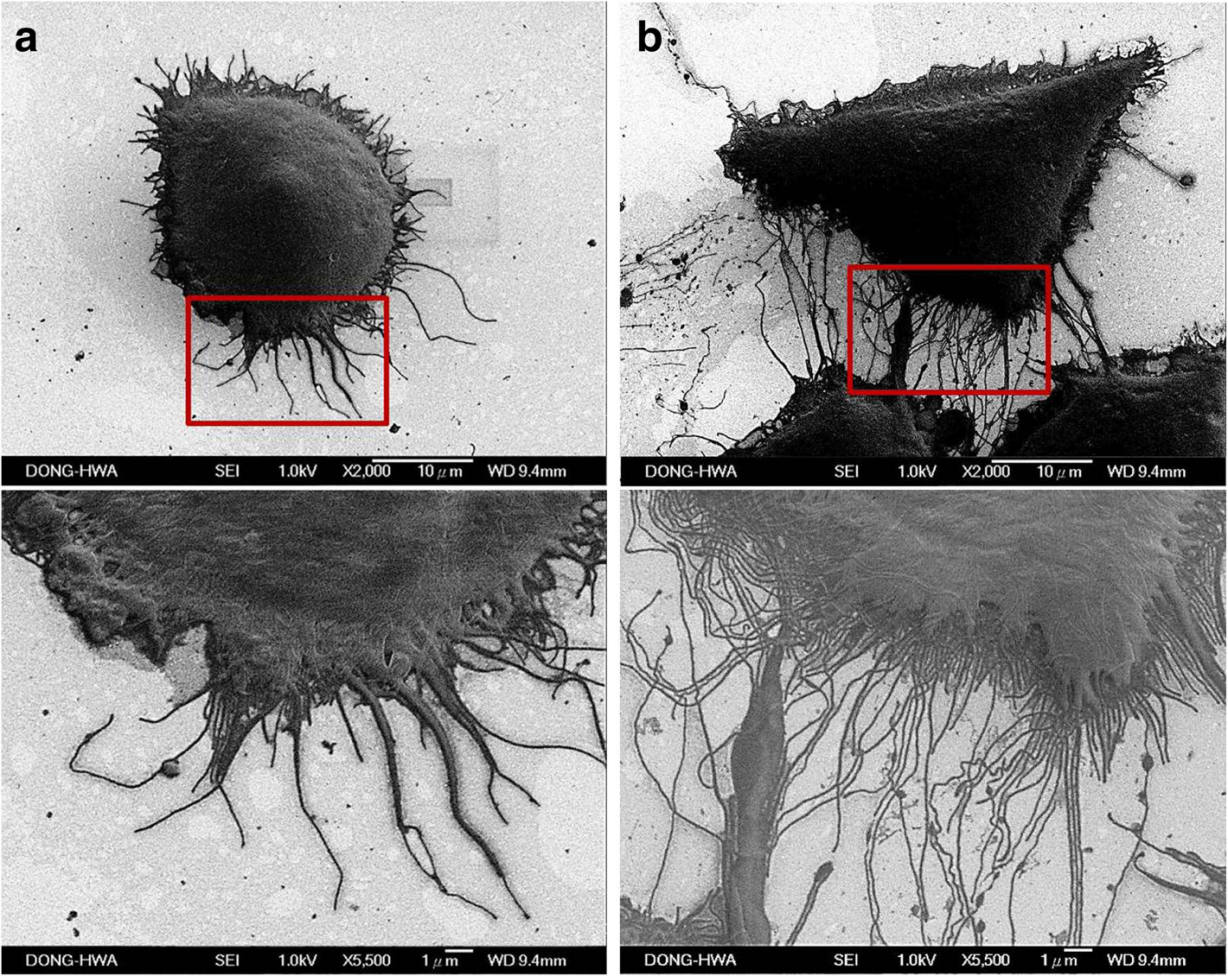
FE-SEM images of A253 cells. Parental A253 (**a**) and A253-5 (**b**) cells were examed under FE-SEM, upper panel show the cell appearance at 2000 X magnificence and lower panel show at 5500 X magnificence. Invasive A253-5 show the flourishing structure of filopodia and lamellipodia

The invasive HNSCC subclones were obtained using the same method described as for an invasion assay. Cells invaded through the membrane of transwell were collected and cultured for another round of selection. Numbers of selection were marked following the name of cells. Parental A253 cultured in low (Fig. 1a) or high density (Fig. 1b) showed mostly epithelia type appearance and A253-3 (Fig. 1c) and A253-5 (Fig. 1d) showed more spindle-like or mesenchymal type appearance (indicated by arrows) under 100X magnificence. Figure 2 shows high-resolution pictures of A253-0 and A253-5 by a FE-SEM. The structure of filopodia and lamellipodia was varied advanced in A253-5, suggesting the better mobility of A253-5 cell.

**Fig. 3:**
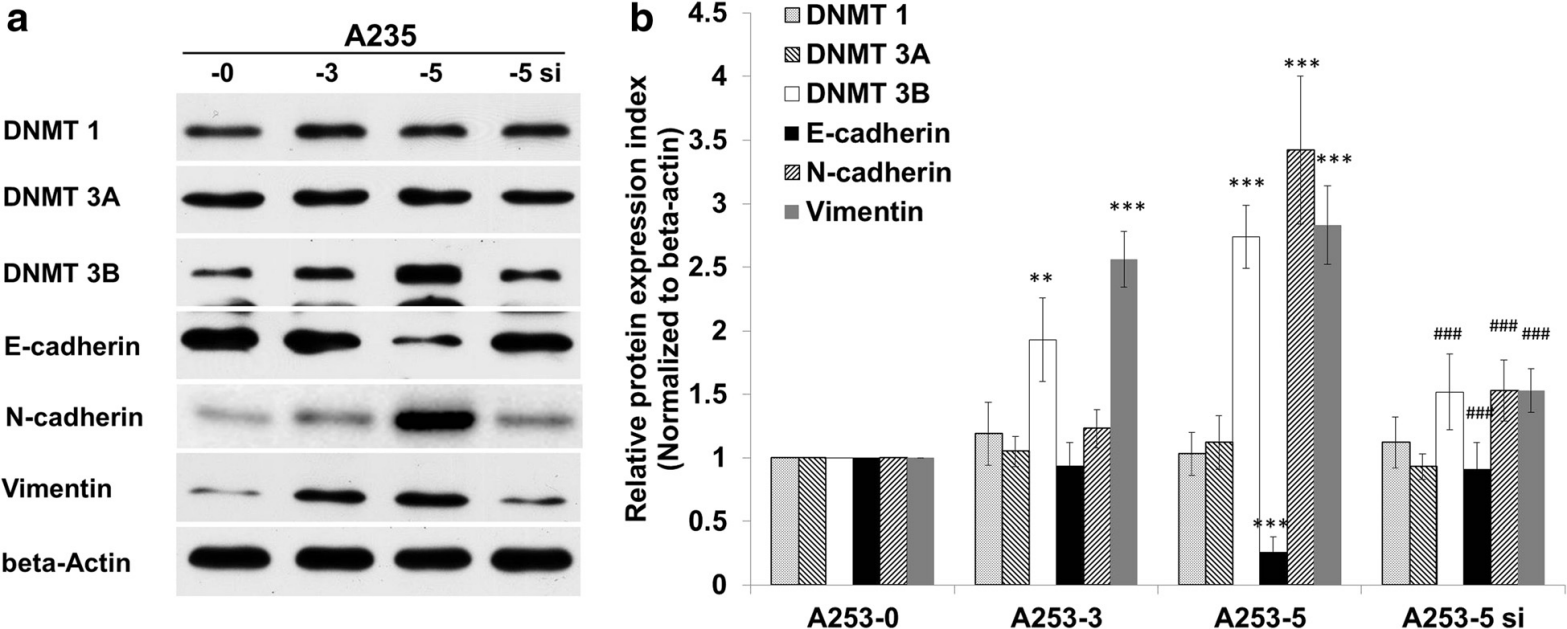
Aberrant expression of DNMT 3B in HNSCC cell lines and knockdown of DNMT 3B in A253-5 reversed EMT marker genes. **a** DNMT 3B protein expression in HNSCC cell lines and its invasive subpopulations. **b** DNMTs and EMT marker genes protein expression in A253 and its invasive subpopulations (A253-3, A253-5) and A253-5 transfected with siRNA against DNMT 3B (A253-5 si). ** indicated *p* < 0.01 and *** indicated *p* < 0.001 as compared with A253-0. ### indicated *p* < 0.001 as compared with A253-5si

DNMT 3B protein expression was aberrant in HNSCC cell lines. Notably, the invasive subclones of A253 and RPMI 2650 had higher expression of DNMT 3B (Fig. 3a) than that of parental cells. In these four HNSCC cell lines, A253 and RPMI2650 also showed the most difference of mobility between parental and filial cells. Moreover, EMT marker genes: E-cadherin (E-cad) was downregulated; N-cadherin (N-cad) and Vimentin were upregulated in A253-5 cell revealed the occurrence of EMT (Fig. 3b). Stable clone of knockdown DNMT 3B was achieved by transfection siRNA against DNMT 3B into A253-5 and marked as A253-5si. Q-PCR results showed the specificity of siRNA (with no influence to DNMT 1 and DNMT 3A) and the knockdown efficiency was around 60 %. Knockdown of DNMT 3B resulted in cell morphology reversion (Additional file 2: Figure S1A, B and C) and up-regulation of E-cad and down-regulation of N-cad and Vimentin, suggesting DNMT 3B may lead to the inhibition of EMT.

### Knockdown of DNMT 3B could restore E-cadherin expression by demethylation of promoter region

**Fig. 4:**
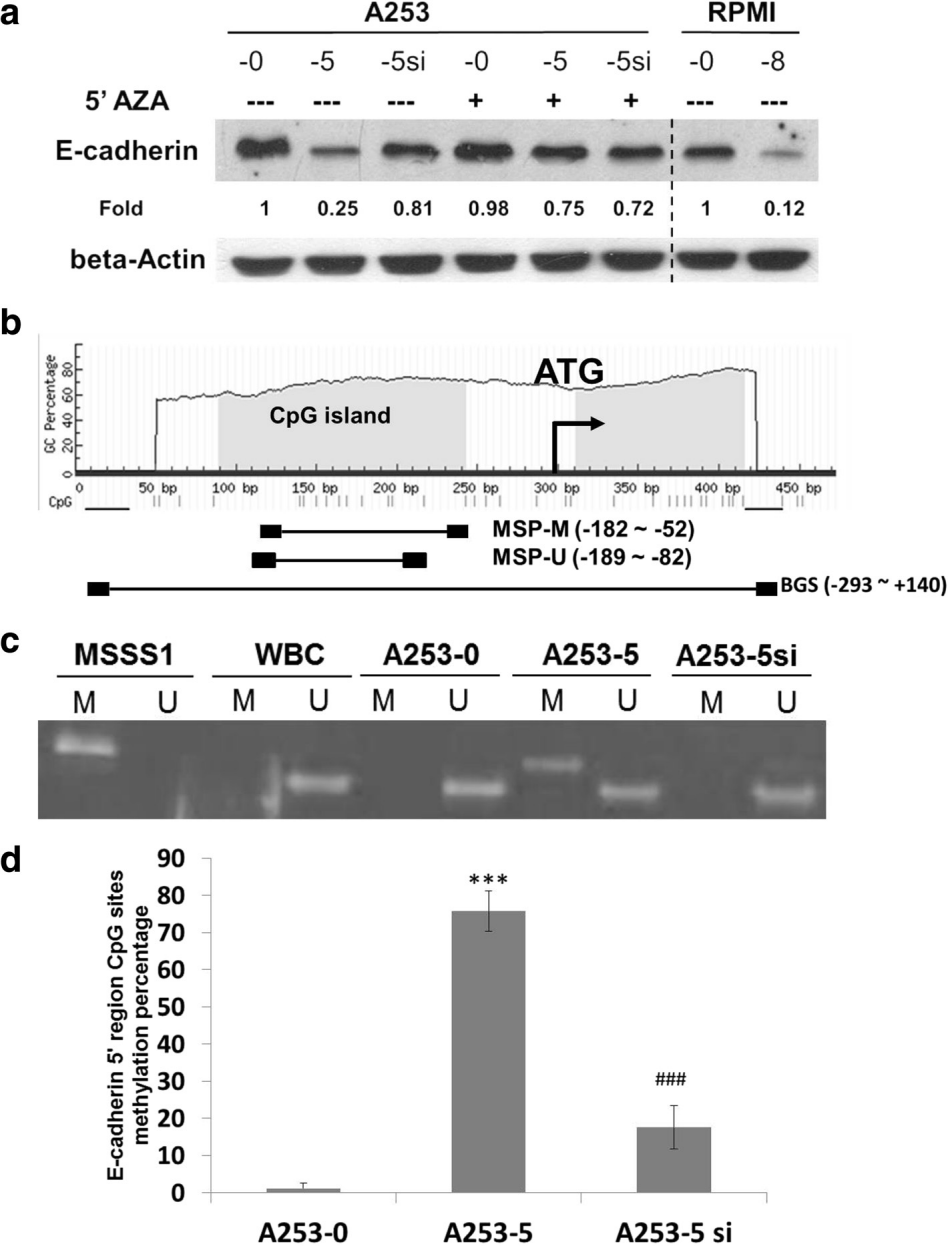
Involvement of DNA methylation in E-cadherin 5′ region. **a** Demethylation agent 5′AZA could restored E-cadherin expression on A253-5 cells. **b** Illustration of CpG islands distribution on 5′ region of E-cadherin. **c** Methylation specific PCR of E-cadherin in A253 cells. M indicated methylated amplicon and U indicated unmethylated amplicon. MSS1, positive control; WBC: normal human white blood cell for negitive control. **d** Bissulfite sequence of E-cadherin in A253 cells reveal that knockdown of DNMT 3B could result in demethylation of 5′ region of E-cadherin. *** indicated *p* < 0.001 as compared with A253-0 and ### indicated *p* < 0.001 as compared with A253-5

5′AZA was applied to inhibit DNMTs activity in A253 cells. The expression of E-cad was restored after 5′AZA treatment in A253-5 suggested that down-regulation of E-cad might be due to promoter methylation (Fig. 4a). The 5′ region of E-cad was analyzed (−300 to +150) and there were 33 CpG sites (Fig. 4b). The MS-PCR results showed the methylated CpG sites in A253-5 and methylated amplicon cannot be detected in A253-5si (Fig. 4c). The BGS results showed that less than 3 % of CpG sites were methylated in parental cells and more than 70 % were methylated in invasive subclone A253-5 (Fig. 4d). These results indicated the knockdown of DNMT 3B caused significantly demethylate of E-cad 5′ region. Photograph of A253-5 cells treated with 5′AZA were taken under 100X magnificence and the cell showed mostly epithelia type appearance (Additional file 2: Figure S1B and D).

### Mir-29b mimic could downregulate DNMT 3B and inhibit EMT

**Fig. 5:**
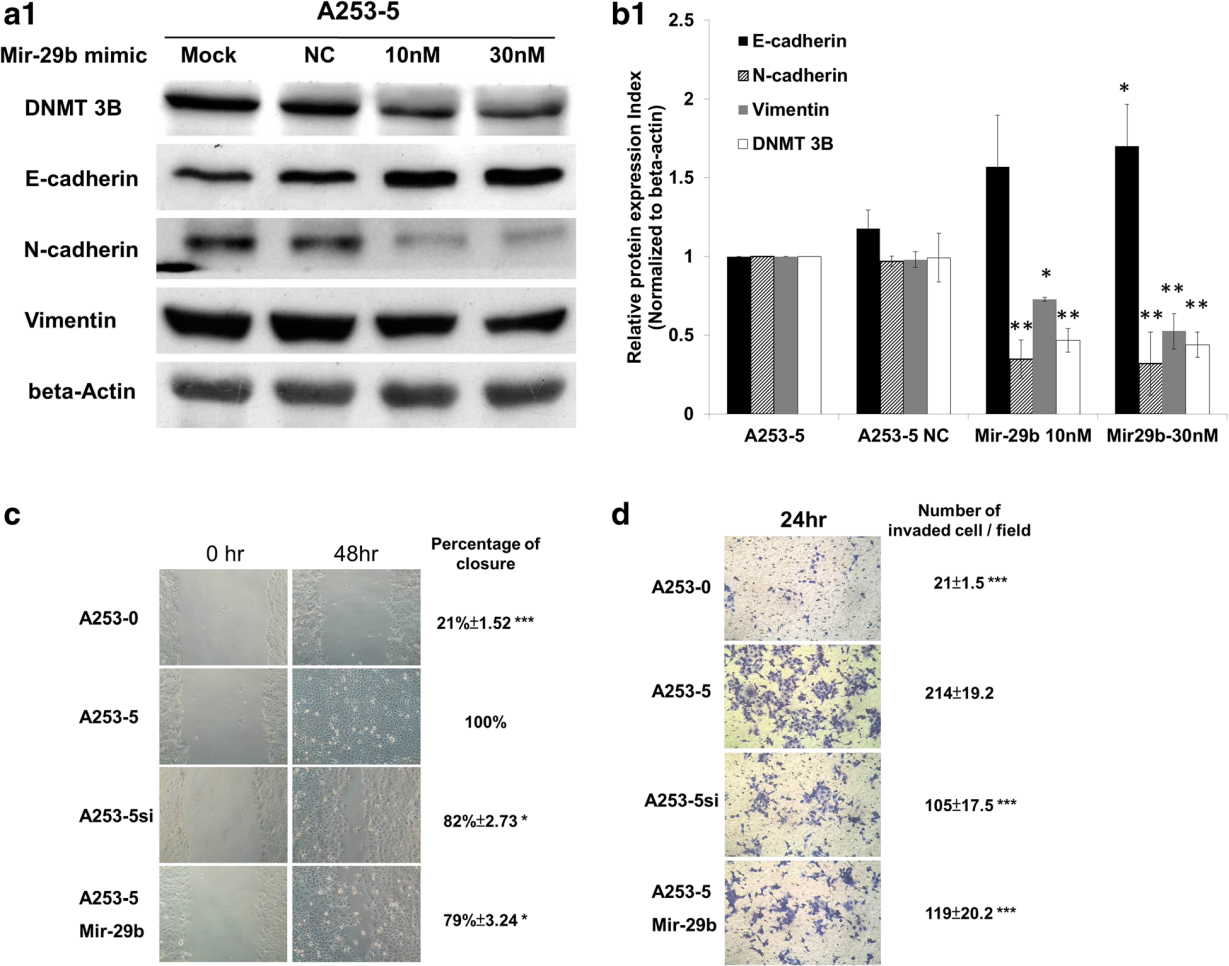
MiRNA 29b mimic targeted DNMT 3B and reversed EMT. **a** Q-PCR examination of miRNA 29b expression in A253 cells. * indicated *p* < 0.05 and ** indicated *p* < 0.01 as compared with A253-0. # indicated that there were no statistically significant between A253-5 and A253-5si (Knockdown of DNMT 3B). **b** Transfection of miRNA 29b mimic could downregulate DNMT 3B and reverse EMT marker genes in A253-5. **c**, **d** Migration and invasion assay. Knockdown DNMT 3B by small interfering RNA or mir-29b mimic could inhibit cell mobility. * indicated *p* < 0.05, ** indicated *p* < 0.01 and *** indicated *p* < 0.001 as compared with A253-5

Interestingly, miRNA 29b was found downregulated in invasive subclones of A253 cells and knockdown of DNMT 3B did not influence the miRNA 29b expression (Fig. 5a). However, A253-5 transfected with miRNA 29b mimic could not only inhibit DNMT 3B expression but also reverse EMT marker genes expression (increase E-cad and decrease N-cad and Vimentin expression; Fig. 4b). Figure 5c and d show that downregulation of DNMT 3B through either small interfering RNA (A253-5si) or miRNA 29b mimic (A253-5 Mir-29b), which could inhibit the migration and invasion of A253-5 in vitro. Photograph of A253-5 cells transfected with miRNA 29b mimic were taken under 100X magnificence and the cell showed mostly epithelia type appearance (Additional file 2: Figure S1B and E).

### mRNA enrichment analysis

A total of 707 genes (412 upregulated and 295 down regulated) were identified with the differentially expression log_2_ |Fold change| ≥ 1 and *p* < 0.05 between A253-0 and A253-5. For the clustering analysis, the first 250 genes with the difference between the maximum and minimum intensity values were selected (Additional file 3: Figure S2). The canonical pathway analysis showed that top 5 pathways (according to most significance in database) are involved in invasive subclone A253-5 cells including arginine and proline metabolism, general pathways in cancer, TGF-beta signaling, focal adhesion and insulin signaling (Table 1). Tables 2, 3 and 4 list individual genes overlapping with three pathways including arginine and proline metabolism, TGF-beta signaling, and focal adhesion, respectively. These results indicated that possible genes or pathways are involved in the induction of EMT and cell invasion.

**Table 1:** Top 5 most enriched pathways of genes differentially expressed in invasive A253 cells according to KEGG pathway enrichment analysis

KEGG pathways	# of genes in overlap/geneset	*P* value
Arginine and proline metabolism	10/54	1.25E-09
Pathways in cancer	21/328	1.48E-09
TGF-beta signaling pathway	10/86	1.29E-07
Focal adhesion	14/201	2.89E-07
Insulin signaling pathway	11/137	1.35E-06

**Table 2:** Differentially expressed genes overlap in KEGG arginine and proline metabolism pathway

Gene_symbol	Description	Log_2_ Ratio (A253-5/A253-0)	*P* value
NOS3	nitric oxide synthase 3	2.7864	1.98847E-18
NOS1	nitric oxide synthase 1	−1.0344	0.001156601
ALDH2	aldehyde dehydrogenase 2 family	−2.2530	1.27408E-16
GLS2	glutaminase 2	−1.1956	0.000792972
GAMT	guanidinoacetate N-methyltransferase	−1.5676	3.38943E-10
GATM	glycine amidinotransferase	1.2157	0.048297875
ODC1	ornithine decarboxylase 1	1.1353	1.1552E-08
P4HA1	prolyl 4-hydroxylase, alpha polypeptide I	−1.0132	0.000552165
PRODH	proline dehydrogenase (oxidase) 1	−1.3810	0.00044575
SAT1	spermidine/spermine N1-acetyltransferase 1	1.0905	0.000181734

**Table 3:** Differentially expressed genes overlap in KEGG TGF-beta signaling pathway

Gene_symbol	Description	Log2 Ratio (A253-5/A253-0)	*P* value
TGFB2	transforming growth factor, beta 2	1.4825	1.26645E-05
SMAD4	SMAD family member 4	−1.8344	7.44456E-12
THBS1	thrombospondin 1	1.0290	0.000135356
BMP7	bone morphogenetic protein 7	−1.0015	1.23496E-05
SMAD6	SMAD family member 6	−1.3877	2.88563E-05
FST	follistatin	1.9942	3.77471E-09
ID1	inhibitor of DNA binding 1	−2.3048	3.76822E-16
ID2	inhibitor of DNA binding 2	−2.7118	5.48439E-12
ID3	inhibitor of DNA binding 3	−2.5933	2.77865E-16
ID4	inhibitor of DNA binding 4	−1.6108	2.7105E-06

**Table 4:** Differentially expressed genes overlap in KEGG focal adhesion pathway

Gene_symbol	Description	Log2 Ratio (A253-5/A253-0)	*P* value
MAPK10	mitogen-activated protein kinase 10	−1.1440	0.000264947
AKT1	v-akt murine thymoma viral oncogene homolog 1	1.1076	0.014492851
PDGFB	platelet-derived growth factor beta polypeptide	1.0834	6.23066E-05
BIRC3	baculoviral IAP repeat containing 3	1.0176	0.001517394
PGF	placental growth factor	−1.0496	0.025969431
COL4A1	collagen, type IV, alpha 1	2.1236	1.58011E-20
FN1	fibronectin 1	3.6490	3.35978E-24
THBS1	thrombospondin 1	1.0290	0.000135356
FLT1	fms-related tyrosine kinase 1	1.2325	0.025381832
RASGRF1	Ras protein-specific guanine nucleotide-releasing factor 1	1.7221	1.11117E-05
CCND2	cyclin D2	2.3619	8.02038E-19
COL5A2	collagen, type V, alpha 2	1.2806	0.030736616
TNC	tenascin C	1.6496	9.83873E-07
PARVA	parvin, alpha	1.2195	9.20291E-07

## Discussion

Accumulating evidence has pointed out the involvement of the EMT in morphological changes from a cobblestone-like of epithelial cells to a spindle-shaped mesenchymal cell morphology [30, 31]. Using the methods descripted in this study, we performed a process of invasion in vitro and obtained the EMT cell with advanced mobility. The appearance of invasive subclone of A253-5 shows advanced structures of filopodia and lamellipodia. These protrusive structures of cell are believed to enforce cells invasion through extracellular matrix (ECM). The major organizer of actin assembly in lamellipodia is the Arp2/3 complex and other subunits (ArpC1 ~ C4) [32]. The Arp2/3 complex initiates the new actin filaments in lamellipodia and is regulated by Scar/WAVE complex [33], which interact with the small GTPase Rac1 [34, 35]. Filopodia are actin-containing spurs, which are involved in cell migration and controlled by numbers of proteins including fascin, diaphanous, and Mena/VASP. Fascin can bundle with actin and form the filament networks to promote filopodia growth [36]. Of note the activity of actin bundling is regulated by small GTPases Rac and Cdc42 [37, 38]. Thereby, the actin dynamics in filopodia and lamellipodia is implicated in the process of cancer metastasis and invasion [32].

DNMTs are enzymes responsible for DNA methylation pattern in cells. However, aberrant DNA methylation of tumor suppressor genes is validated as a frequent molecular event in human carcinomas [20]. Aberrant DNMT 3B expression is associated with various cancers including breast cancer [39], colorectal cancer [40, 41], stomach cancer [40, 41], and lung cancer [42]. It is also suggested that DNMT 3B is required for tumor development [43] and promotes tumorigenesis by hypermethylation of tumor suppresser gene such as Sfrp family [44]. Moreover, the transcription variants of DNMT 3B have shown its role in tumor progression [45]. There are seven aberrant transcripts from unconventional pre-mRNA splicing found in lung cancer. One of the transcription variant, DNMT 3B7, is demonstrated by the effect on E-cad methylation and 2-fold decrease in E-cad expression paralleled [46]. DNMT3B7 has recently shown its promoting role in tumor progression to a more invasive phenotype in breast cancer cell lines [47].

Previous evidence has shown that the down regulation of mir-29b is found in non-small cell lung cancer [48], glioblastoma [49], prostate cancer [50], ovarian cancer [51], and HNSCC [52]. Several lines of cellular function in mir-29b are proposed such as promotion of apoptosis, suppression of tumor invasion, and regulation of EMT. Firstly, miR-29 family can trigger cell apoptosis by directly binding to Mcl-1(anti-apoptotic gene) and preventing expression of Mcl-1 [53]. Enhancements of the miR-29b expression may reduce Mcl-1 protein and increase the cytotoxicity induced by tumor necrosis factor-related apoptosis-inducing ligand (TRAIL) [54]. In acute myeloid leukemia (AML) cell, ectopic transfection of synthetic miR-29b can up-regulate the pro-apoptotic genes, such as BIM (BCL2L11) and the tumor suppressor programmed cell death-4 (PDCD4) [55]. Secondly, the inhibition of miR-29b causes the increasing expression of DNA binding 1 (ID1) and MMP9, which lead to tumor cell invasion [56]. MiR-29 family has also demonstrated its tumor suppressor role by targeting laminin γ2 (LAMC2) and α6 integrin (ITGA6) resulting in the inhibition of cell migration and invasion in HNSCC cell line (SAS and FaDu) [52]. Thirdly, overexpression of miR-29 may block EMT by targeting ADAM12, which is highly associated with invasion and EMT in breast cancer [57]. Moreover, a transcription factor GATA3 is found to promote miR-29b expression and suppress metastasis of breast cancer by inducing miR-29b. MiR-29b may target numbers of pro-metastatic regulators associated with angiogenesis, collagen remodeling and proteolysis; thereby, altering the tumor microenvironment [58, 59]. Furthermore, miR-29 family is reported its involvement in the downregulation of DNMT 3A and 3B in lung cancer cell line and AML, suggesting that miR-29b may alter the methylation status of tumor-suppressor genes resulting in the inhibition of carcinogenic process [48, 60].

According the mRNA enrichment analysis, we reported the most involvement of five pathways in invasive A253 cell (Table 1). We focus on three of pathways including arginine and proline metabolism, TGF-beta and focal adhesion with discussion. Proline dehydrogenase (PRODH) is a mitochondrial enzyme transferring the electrons from proline and producing pyrroline-5-carboxylate (P5C). A hypothetical proline metabolic timeline in cancer was also proposed [61]. During the early stage of cancer progression like chronic inflammation and DNA damage, PRODH could be induced by PPARγ [62] and P53 [63], respectively. PRODH was reported as a tumor suppressor gene in 2009 [64]. Using the Tet-off system, the growth of xenograft colorectal tumor is suppressed by expressing PRODH in immune-deficient mice. The immunohistochemical (IHC) assay was also applied to examine the expression of PRODH in digestive tract and renal tumor. Comparing to the normal tissue, 78 and 85 % of the tumors (digestive tract and renal tumor, respectively) have markedly decreased or undetectable PRODH expression [64, 65]. From our results, PRODH was found 2.72 fold down regulated in invasive A253-5 cell.

TGF-beta displays a distinct role in tumor development [66]. In the initial stage of tumor progression, TGF-beta may induce growth arrest and apoptosis onto cancer cells. However, TGF-beta plays as a tumor promoter role due to the induction of EMT and increasing of invasiveness in late stages [67]. Interestingly, SMAD4 is the key mediator of TGF-beta signaling [68] and is found 3.67 fold down regulated in invasive A253-5 cell. Previous studies have shown that a loss of SMAD4 expression by deletion in mice model might trigger spontaneous HNC, mammary gland tumors, and skin SCC development [69–71]. The genome wide analysis of HNSCC also showed the frequent deletion of SMAD4 [72] and heterozygous loss of Smad4 in HNSCC [73]. The reduction of SMAD4 is found in tumors, 67 % of the adjacent non-tumor tissues from HNSCC patients also show more than 50 % SMAD4 reduction [71]. These results imply that the down regulation of SMAD4 might be an early event in HNSCC progression. A recent study shows that TGF-beta could induce EMT and change DNA methylation status by upregulated DNMTs in ovarian cancer cells [74]. Considering the downregulation of SMAD4, SMAD6, and the inhibitor of DNA binding family (ID1 ~ ID4), the involvement of TGF-beta in our model system may be through SMAD-independent or non-SMAD signaling [75]. Par6 may be phosphorylated by activated TGF-beta receptor II and regulate Rho GTPase activity resulting in actin polymerization and negatively regulation of tight junction assembly during the EMT [76]. Moreover, the ubiquitin ligase TRAF6 may be activated by TGF-beta receptor I following the activation of JNK and p38 MAP kinases leading to EMT [77].

Most of genes identified in the focal adhesion pathway are upregulated and indicated a positive regulation of mobility in invasive A253-5 cell. Fibronectin (FN) is a well-defined mesenchymal marker [10] and is found 12 fold upregulated in invasive A253-5 cell. FN is involved in many cellular functions including cell migration, differentiation, wound healing, and carcinoma development [78–80]. During the EMT, epithelia cell loss of the cell-cell contact and switch to cell-ECM interaction implicit the possibility of EMT promoting role of FN. Cultured breast cancer cells with FN induced EMT through Scr and ERK/MAP kinases pathways support a high level of FN, which was detected in the breast tumor sections [81]. Collagen type IV alpha 1 (COL4A1), another mesenchymal marker gene [18] was identified with 4.42 fold upregulated in invasive A253-5 cell. A recent study showed that the knockdown of COL4A1 could reduce mouse melanoma cell motility and decrease lung metastasis in vivo [82]. Tenascin C (TNC) is a target gene of TGF-beta [83] and is found 3.04 fold upregulated in invasive A253-5 cell. TNC is an expression exerted as the invasive edge of breast tumor and serves as a prognostic marker for local and distant recurrence [84]. TNC is also highly expressed in the microenvironment of most solid tumors by fibroblast cells [85] as a pro-invasive signal leading to cell invasion through activation of RhoA and Rac [86]. Moreover, co-expression of TNC and vimentin might induce mesenchymal-like phenotype in breast cancer cell [87].

## Conclusion

Here we report the involvement of DNMT 3B in the induction of EMT of HNSCC cell lines. The downregulation of DNMT 3B by miR-29b mimic may reverse EMT and inhibit cell migration and invasion. Gene expression profile of invasive HNSCC is also presented. Using a bioinformatics tool, we are able to identify various genes that participated in metabolism and cancer related pathways, which may be involved in HNSCC metastasis. Although these findings require further experimental validations, follow-up research could find new molecules that could serve as prognostic factors or therapeutic targets for invasive HNSCC.

## Abbreviations

5′AZA, 5-aza-2′-deoxycytidine; BGS, bisulfite genomic sequencing; E-cad, E-cadherin; EMT, epithelial–mesenchymal transition; FBS, Fetal bovine serum; HNSCC, head and neck squamous cell carcinoma; MS-PCR, methylation-specific polymerase chain reaction; N-cad, N-cadherin; PVDF, polyvinylidene difluoride; siRNA, small interfering RNA

## Additional files

Additional file 1: Table S1. Primers used in this study. Primers list for MS-PCR, MSG and Q-PCR. (DOCX 14 kb) Additional file 2: Figure S1. Morphology changed of various treatments of A253 cells. A253-0 cell (A), A253-5 (B), A253-5si (C), A253-5 treated with 5′AZA (D) and A253-5 transfected with miRNA29b mimic (E). Photographs were taken at 100 X magnificence and mesenchymal type cells were indicated by arrows. (DOCX 430 kb) Additional file 3: Figure S2. Clustering analysis of difference genes expression between A253-0 and A253-5. A total of 250 genes with the difference between the maximum and minimum intensity values were clustered. (JPG 172 kb)
