# Erratum to: Involvement of DNMT 3B promotes epithelial-mesenchymal transition and gene expression profile of invasive head and neck squamous cell carcinomas cell lines

**DOI:** 10.1186/s12885-016-2560-2

**Published:** 2016-08-03

**Authors:** Li-Hsuen Chen, Wen-Lin Hsu, Yen-Ju Tseng, Dai-Wei Liu, Ching-Feng Weng

**Affiliations:** 1 Department of Life Science and the Institute of Biotechnology, National Dong Hwa University, Hualien, Taiwan; 2 Department of Radiation Oncology, Buddhist Tzu Chi General Hospital, Hualien, Taiwan; 3 School of Medicine, Tzu Chi University, Hualien, Taiwan

## Erratum

After publication of the original article [1], the authors noticed an error within Figs. 3 and 5: Figures 3a and 5a were inadvertently missed. The full versions of Figs. 3 and 5 have been updated in the original article and can be found below. We would like to apologise for the error and for any inconvenience this may have caused.

**Fig. 3:**
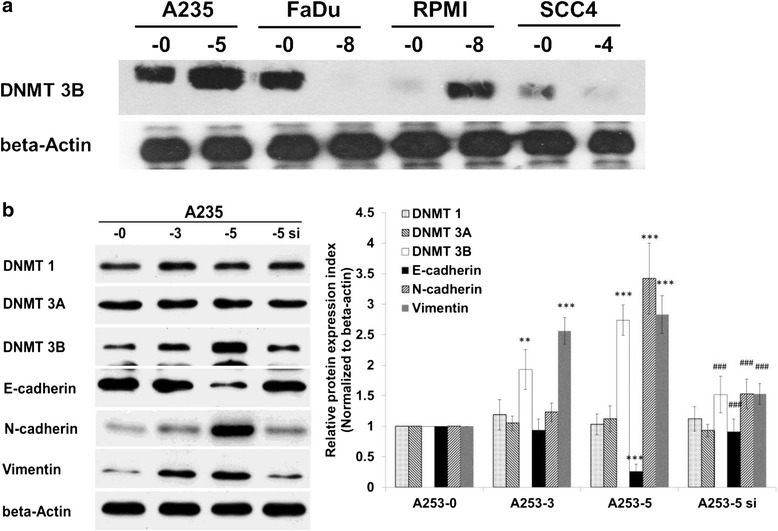
Aberrant expression of DNMT 3B in HNSCC cell lines and knockdown of DNMT 3B in A253-5 reversed EMT marker genes. **a** DNMT 3B protein expression in HNSCC cell lines and its invasive subpopulations. **b** DNMTs and EMT marker genes protein expression in A253 and its invasive subpopulations (A253-3, A253-5) and A253-5 transfected with siRNA against DNMT 3B (A253-5 si). ** indicated *p* < 0.01 and *** indicated *p* < 0.001 as compared with A253-0. ### indicated *p* < 0.001 as compared with A253-5si

**Fig. 5:**
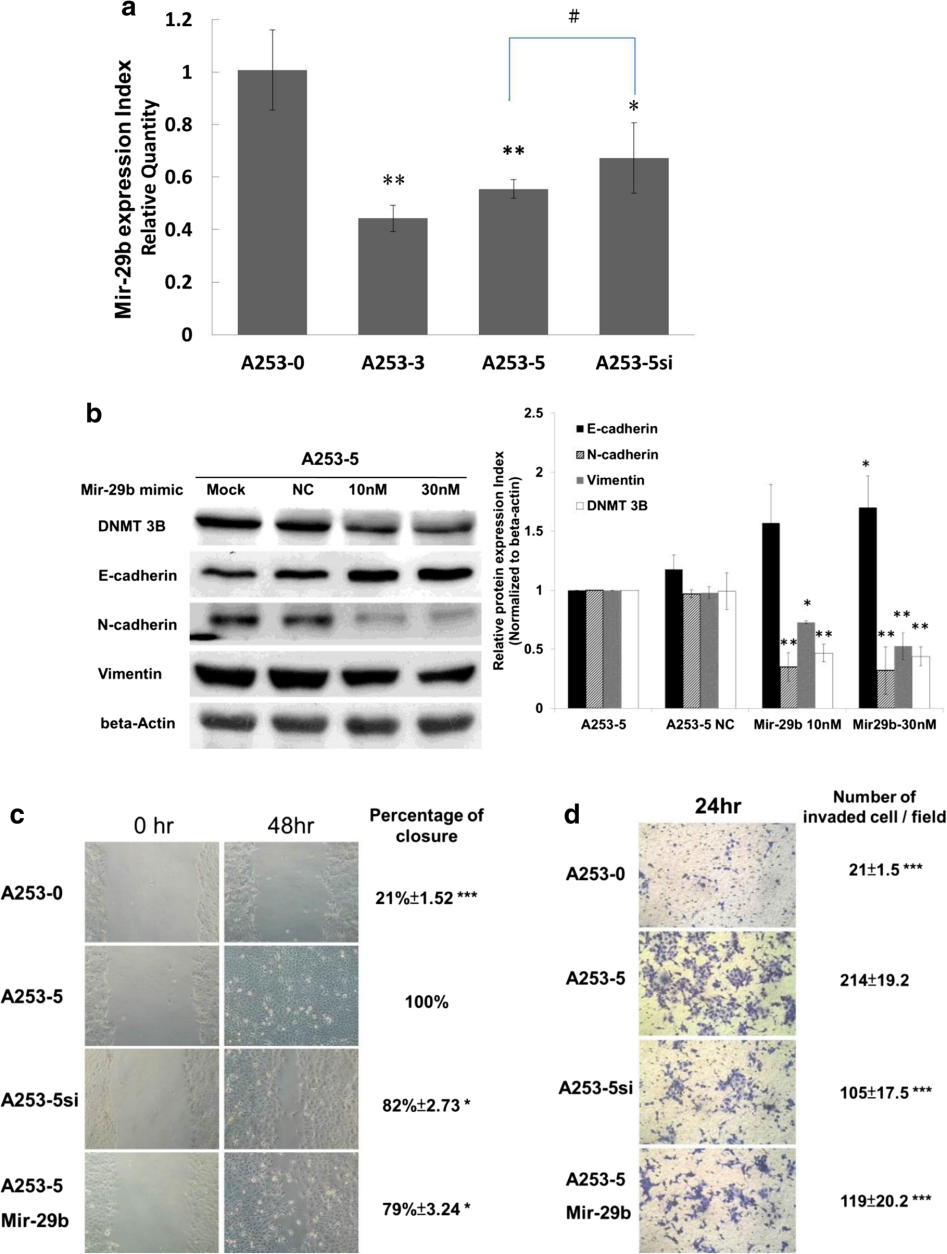
MiRNA 29b mimic targeted DNMT 3B and reversed EMT. **a** Q-PCR examination of miRNA 29b expression in A253 cells. * indicated *p* < 0.05 and ** indicated *p* < 0.01 as compared with A253-0. # indicated that there were no statistically significant between A253-5 and A253-5si (Knockdown of DNMT 3B). **b** Transfection of miRNA 29b mimic could downregulate DNMT 3B and reverse EMT marker genes in A253-5. **c**, **d** Migration and invasion assay. Knockdown DNMT 3B by small interfering RNA or mir-29b mimic could inhibit cell mobility. * indicated *p* < 0.05, ** indicated *p* < 0.01 and *** indicated *p* < 0.001 as compared with A253-5
