# Supplementary material for: Involvement of DNMT 3B promotes epithelial-mesenchymal transition and gene expression profile of invasive head and neck squamous cell carcinomas cell lines
Source: BMC Cancer. 2016 Jul 8;16:431. doi: 10.1186/s12885-016-2468-x (PMC4938990; doi:10.1186/s12885-016-2468-x)
Supplement: Additional file 1: Table S1. — Primers used in this study. Primers list for MS-PCR, MSG and Q-PCR. (DOCX 14 kb) [file 12885_2016_2468_MOESM1_ESM.docx]

**Supplemental** Table 1. Primers used in this study.

| **Genes** | **Sequence** |  |
| --- | --- | --- |
| **E-cad MSP-M** | F 5’ TTAGGTTAGAGGGTTATCGCGT 3’  R 5’ TAACTAAAAATTCACCTACCGAC 3’ | |
| **E-cad MSP-U** | F 5’ TAATTTTAGGTTAGAGGGTTATTGT 3’  R 5’ CACAACCAATCAACAACACA 3’ | |
| **E-cad MSG** | F 5’ TAAGTGTAAAAGTTTTTTTTGATTTTA 3’  R 5’ ACTCCAAAAACCCATAACTAACC 3’ | |
| **Snail** | F 5’ AATCGGAAGCCTAACTACAGCG 3’  R 5’ GGTCCCAGATGAGCATTGGCA 3’ |  |
| **Slug** | F 5’ AAGCATTTCAACGCCTCCAAA 3’  R 5’ AGGATCTCTCTGGTTGTGGTATGAG 3’ | |
| **Twist** | F 5’ AGCTACGCCTTCTCGGTCT 3’  R 5’ TCCTTCTCTGGAAACAATGACA 3’ |  |
| **GADPH** | F 5’ ACCCACTCCTCCACCTTTGACG 3’  R 5’ TCTCTTCCTCTTGTGCTCTTG 3’ |  |
