# Supplementary material for: Involvement of DNMT 3B promotes epithelial-mesenchymal transition and gene expression profile of invasive head and neck squamous cell carcinomas cell lines
Source: BMC Cancer. 2016 Jul 8;16:431. doi: 10.1186/s12885-016-2468-x (PMC4938990; doi:10.1186/s12885-016-2468-x)
Supplement: Additional file 2: Figure S1. — Morphology changed of various treatments of A253 cells. A253-0 cell (A), A253-5 (B), A253-5si (C), A253-5 treated with 5′AZA (D) and A253-5 transfected with miRNA29b mimic (E). Photographs were taken at 100 X magnificence and mesenchymal type cells were indicated by arrows. (DOCX 430 kb) [file 12885_2016_2468_MOESM2_ESM.docx]

**Supplemental Figure 1.** Morphology changed of various treatments of A253 cells. A253-0 cell (A), A253-5 (B), A253-5si (C), A253-5 treated with 5’AZA (D) and A253-5 transfected with miRNA 29b mimic (E). Photographs were taken at 100 X magnificence and mesenchymal type cells were indicated by arrows.


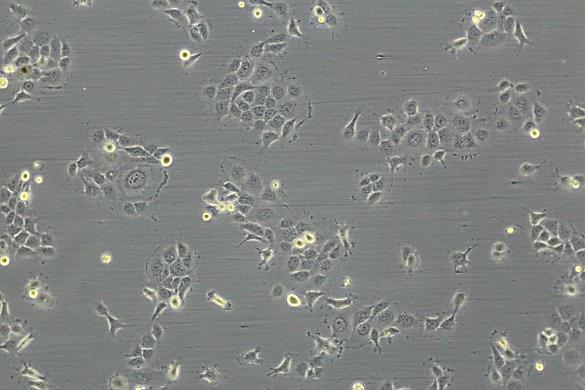

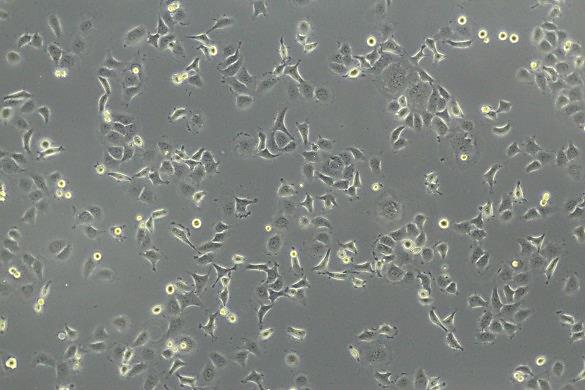


**B**

**A**


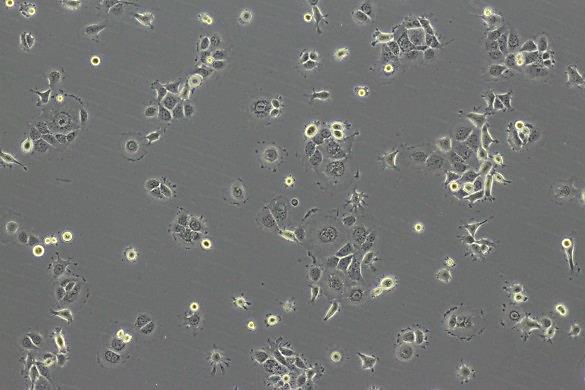

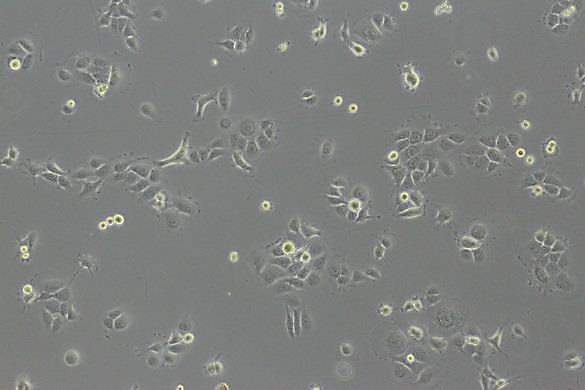


**D**

**C**


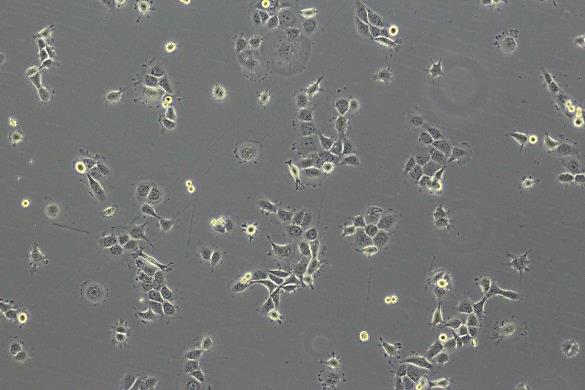


**E**
