# Supplementary figures and images for: Involvement of DNMT 3B promotes epithelial-mesenchymal transition and gene expression profile of invasive head and neck squamous cell carcinomas cell lines
Source: BMC Cancer. 2016 Jul 8;16:431. doi: 10.1186/s12885-016-2468-x (PMC4938990; doi:10.1186/s12885-016-2468-x)

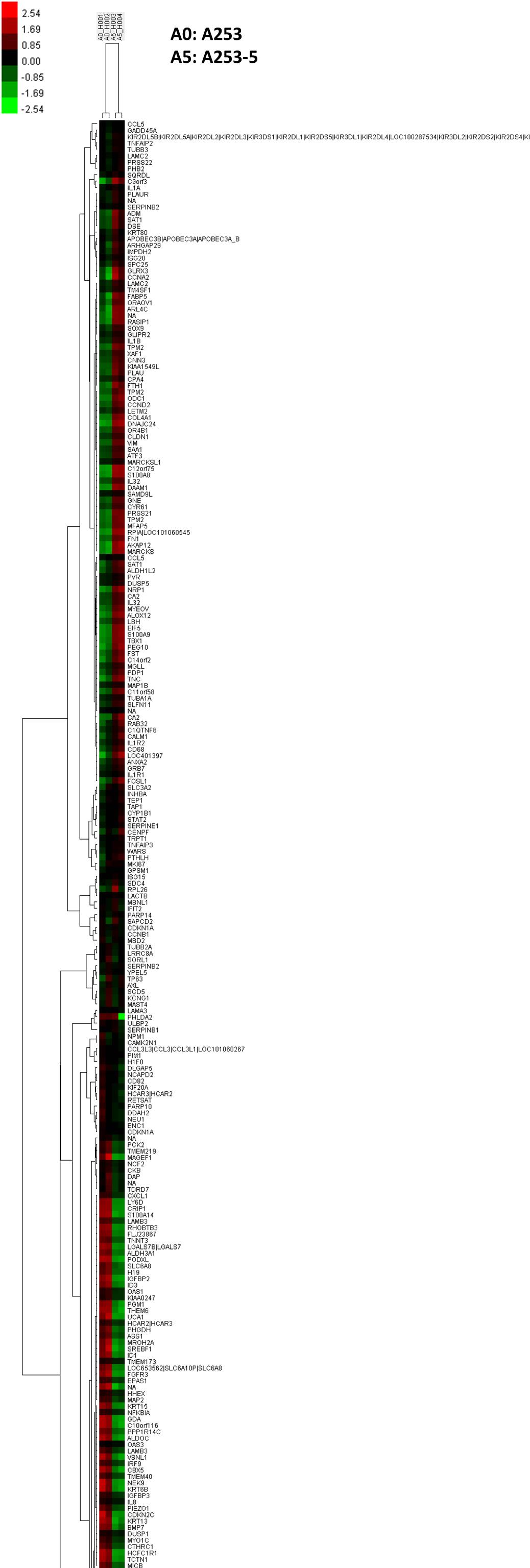

Supplement: Additional file 3: Figure S2. — Clustering analysis of difference genes expression between A253-0 and A253-5. A total of 250 genes with the difference between the maximum and minimum intensity values were clustered. (JPG 172 kb) [file 12885_2016_2468_MOESM3_ESM.jpg]
